# Robotic Resection of Colonic Medullary Carcinoma: A Case Report

**DOI:** 10.7759/cureus.79624

**Published:** 2025-02-25

**Authors:** Justin M Hsieh, Joy Chakraborty

**Affiliations:** 1 Department of Surgery, Greenslopes Private Hospital, Brisbane, AUS

**Keywords:** colorectal medullary carcinoma, microsatellite instability, minimally invasive surgery, mismatch repair deficiency, robotic colorectal surgery

## Abstract

Colorectal medullary carcinoma (MC) is a rare and distinct subtype of colorectal cancer associated with mismatch repair (MMR) deficiency with the loss of MLH1 and PMS2 genes. Due to its rare incidence and atypical histopathological features, MC is often misclassified as a poorly differentiated adenocarcinoma. We present the case of an 87-year-old elderly male with iron deficiency anemia and a right-sided abdominal mass. Initial colonoscopy biopsy reported a poorly differentiated adenocarcinoma, but subsequent surgical resection confirmed MC. The patient elected to undergo a right hemicolectomy via a robotic-assisted approach. Although the potential oncologic benefit of robotic surgery remains uncertain, it was chosen in this case due to the patient’s preference for a minimally invasive procedure, his advanced age, and the tumor’s relatively large size. No adjuvant therapy was recommended after a multidisciplinary review given the tumor’s MMR deficiency and absence of regional lymph node metastasis. This report highlights the diagnostic challenges of MC and the potential benefits of a robotic approach for MC resections in an elderly patient.

## Introduction

Colorectal medullary carcinoma (MC) is a rare entity, representing approximately 0.03% of all colorectal malignancies [1]. Despite its high-grade morphology, MC exhibits unique histopathological and immunohistochemical features such as solid, trabecular architecture, marked lymphocytic infiltration, and frequent loss of mismatch repair (MMR) proteins (i.e., MLH1 and PMS2 genes), often leading to microsatellite instability (MSI) [2,3]. This distinct histological profile can represent a paradoxically favorable prognosis compared to other high-grade colorectal cancers [4]. However, MC’s rarity and histological overlap with poorly differentiated adenocarcinoma frequently result in its misclassification, which complicates optimal management. Standard curative treatment for localized colon cancer is surgical resection, yet the role of adjuvant chemotherapy in MC remains controversial, given its exclusion from large-scale clinical trials [5]. While both laparoscopic and robotic approaches are recognized as minimally invasive alternatives to open surgery, few reports have evaluated the specific utility of robotic-assisted resection for MC. In this case, we detail the presentation, diagnostic evaluation, and surgical management of an 87-year-old patient with MC of the ascending colon. We also highlight the rationale for a robotic approach and its potential benefit with the patient’s postoperative recovery course.

## Case presentation

**Figure 1:**
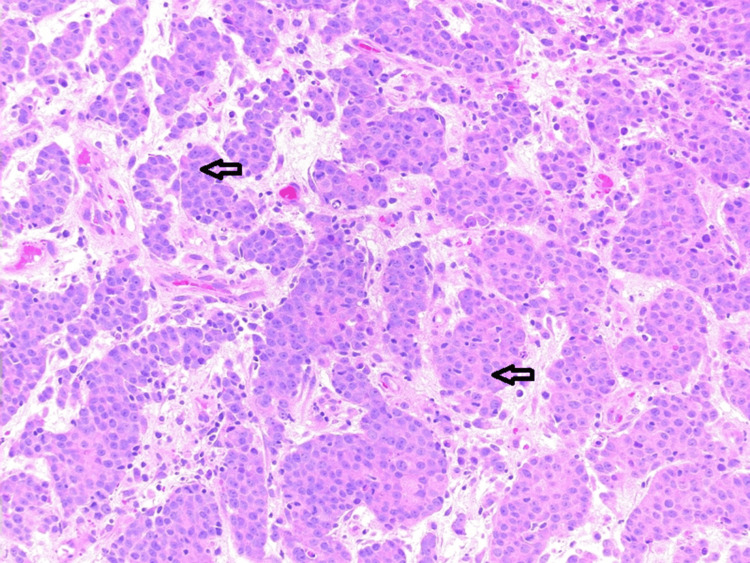
Hematoxylin and eosin (H&E) stain of the colonoscopic biopsy. Arrows indicate areas of high-grade nuclear pleomorphism and scant glandular formation, initially interpreted as poorly differentiated adenocarcinoma.

An 87-year-old male, living independently at home with mild hypertension and dyslipidemia, presented with unexplained iron deficiency anemia. He reported no changes in bowel habits or overt gastrointestinal symptoms, but a physical examination revealed a palpable mass in the right flank. A colonoscopy demonstrated a partially obstructing lesion in the ascending colon, and a biopsy initially reported a poorly differentiated adenocarcinoma (Figure 1).

**Figure 2:**
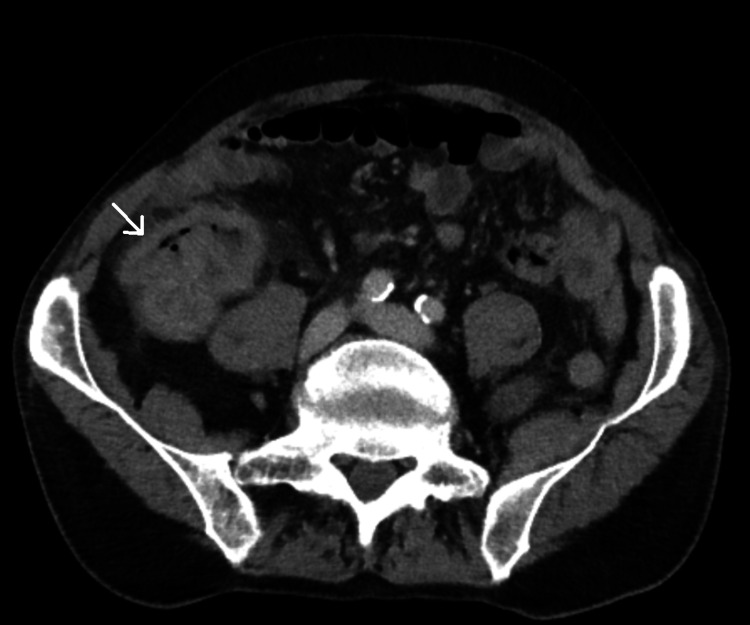
Axial CT scan of the abdomen showing the ascending colon mass. White arrow indicating the location of the ascending colon tumor. CT, computed tomography

**Figure 3:**
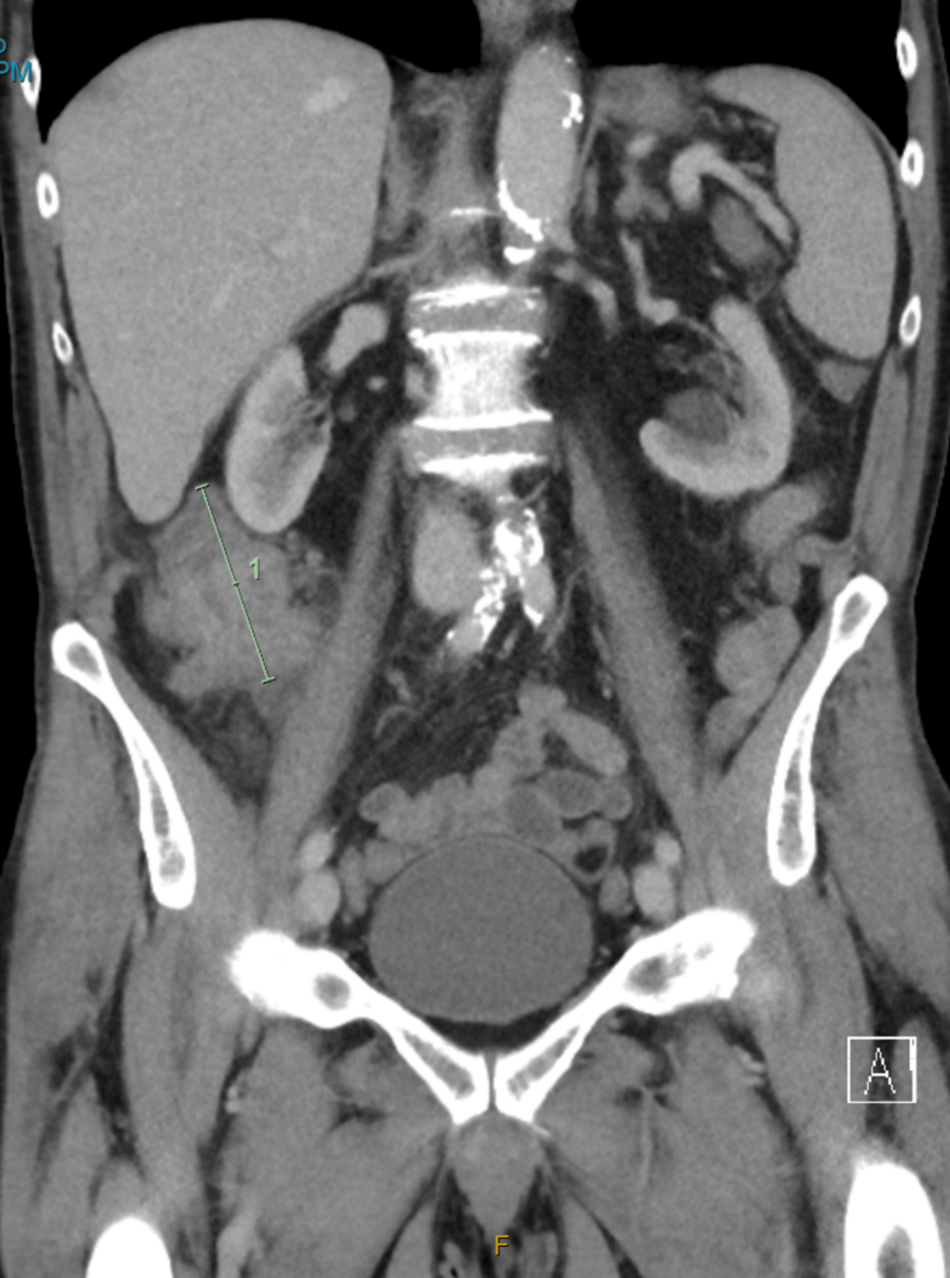
Coronal CT reconstruction indicating the tumor’s longitudinal extent. A green line indicating the location and length of the ascending colon tumor (1 unit length = 68 mm).

Contrast-enhanced CT of the abdomen and pelvis confirmed a large (68 mm) ascending colon tumor with no evidence of nodal or distant metastases (Figures 2, 3). The patient’s carcinoembryonic antigen (CEA) level was within normal limits, and a mild elevation of C-reactive protein (CRP) was noted. No other significant laboratory abnormalities were observed.

The decision to proceed with a robotic-assisted right hemicolectomy was based on several patient‐specific and tumor‐related considerations. First, the patient expressed a clear preference for a minimally invasive operation, valuing a faster recovery and shorter hospital stay. Given his advanced age and functional independence, it was particularly important to reduce the physiologic impact of surgery. Second, the tumor’s large size (68 mm) and partial luminal obstruction required careful dissection around the tumor for a complete surgical resection without involved margins. The articulated instruments and three‐dimensional imaging provided by the robotic platform supported an advantageous dissection around a large bulky tumor. This influenced the surgical team’s choice of a robotic approach with its enhanced visualization and maneuverability compared to conventional laparoscopic techniques. 

**Figure 4:**
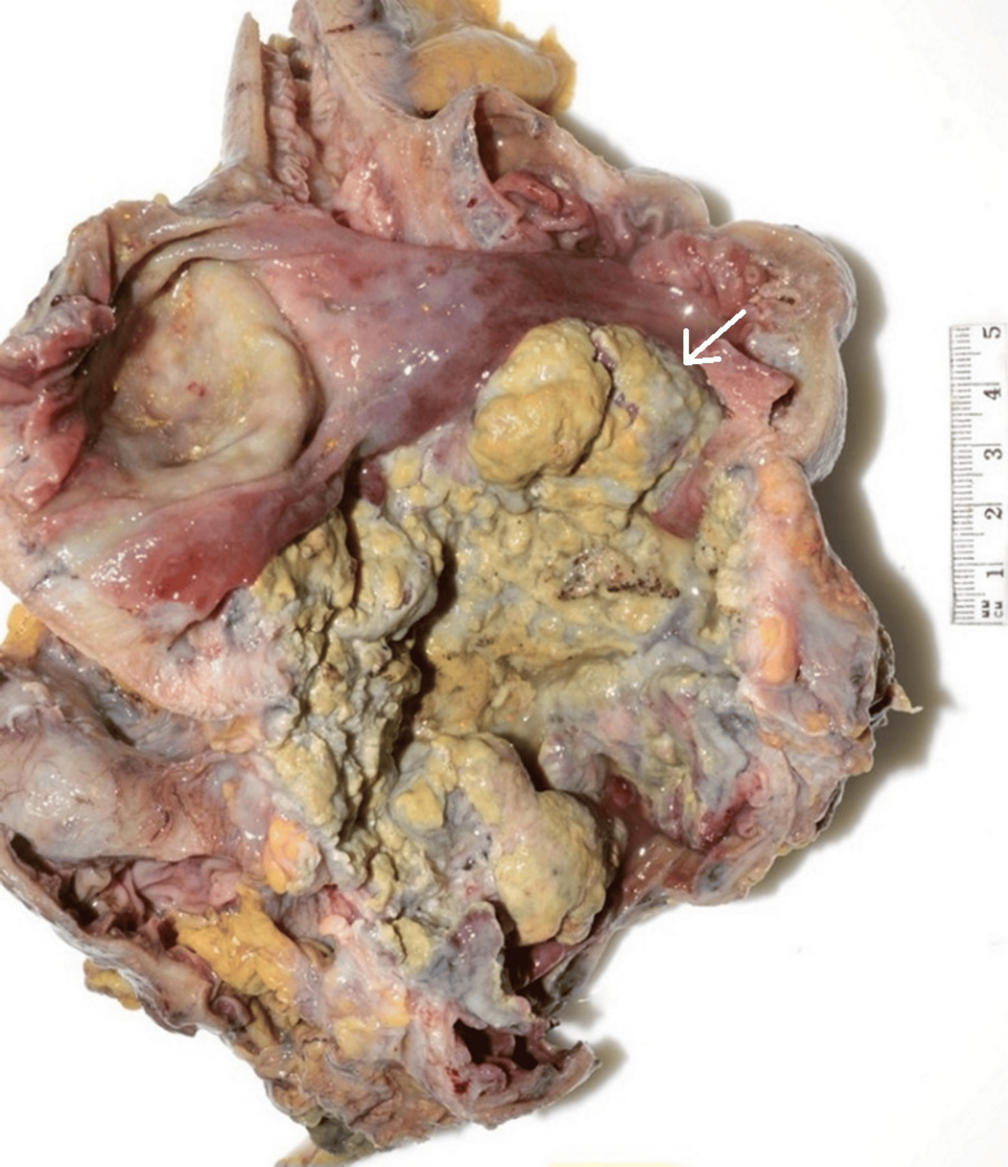
Gross specimen of the ascending colon after resection. The white arrow highlights the tumor. The scale bar on the right indicates size in centimeters (cm).

During the operation, a large, firm mass was seen to be confined in the ascending colon (Figure 4). No evidence of peritoneal carcinomatosis or nodal enlargement was identified, and the small bowel and the rest of the colon appeared normal. A robotic-assisted right hemicolectomy with a stapled ileocolic anastomosis was performed without complication.

**Figure 5:**
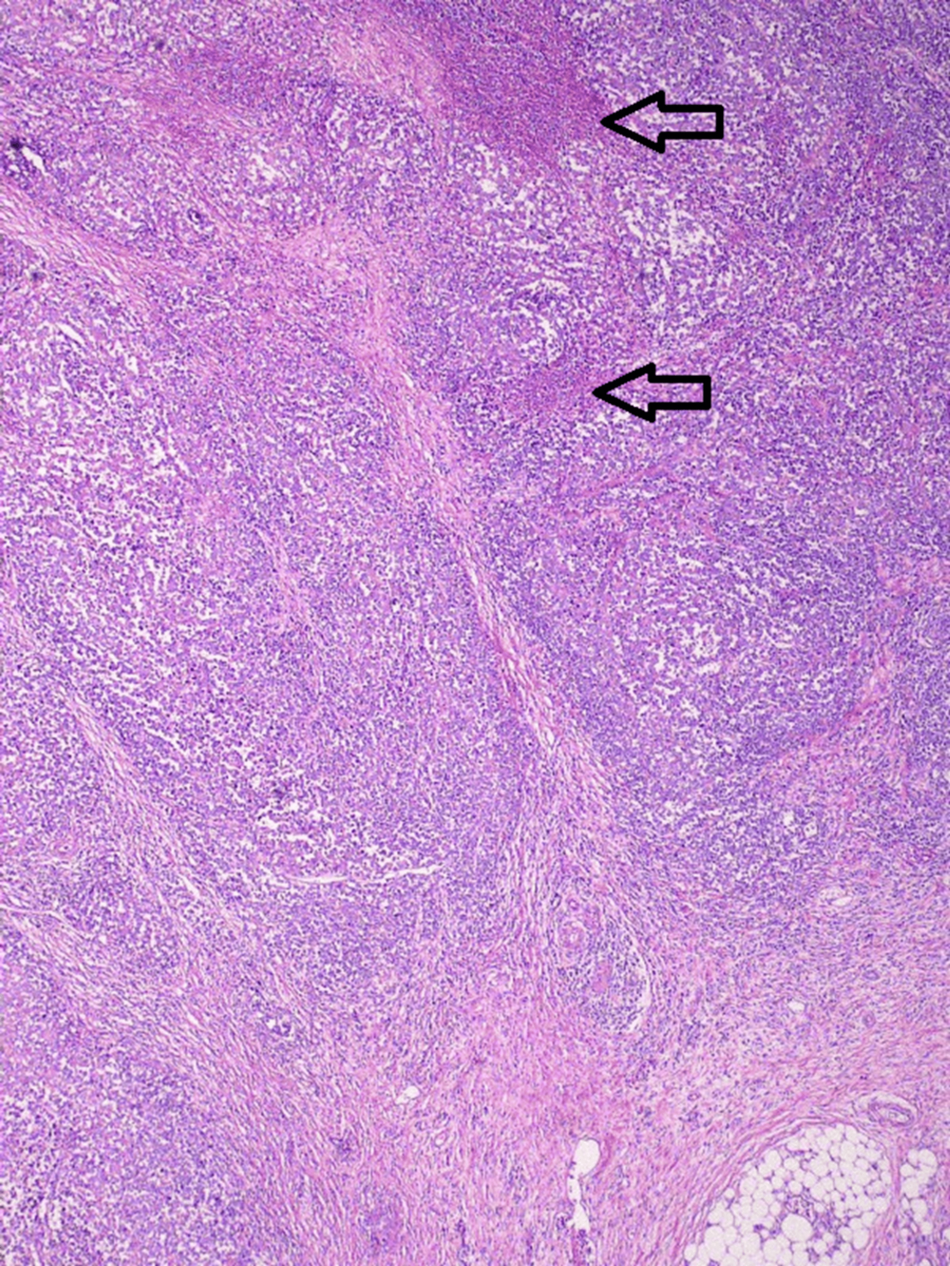
Representative hematoxylin and eosin (H&E) stain of the resected specimen. Arrows mark areas of dense lymphocytic infiltration with polygonal tumor cells.

Histological examination of the resected specimen confirmed a poorly differentiated carcinoma displaying the hallmark features of medullary carcinoma, including a solid, trabecular growth pattern and marked lymphocytic infiltration (Figure 5). The tumor infiltrated the pericolic adipose tissue, thus meeting the criteria for T4 disease, but all 20 sampled lymph nodes were negative for metastasis (N0), consistent with stage IIA disease. Although lymphovascular invasion was present, there was no evidence of perineural invasion. Immunohistochemistry revealed loss of MLH1 and PMS2, with intact MSH2 and MSH6, indicating MMR deficiency and MSI. Given these findings and the absence of nodal involvement, the local colorectal multidisciplinary team concluded that adjuvant chemotherapy would likely provide minimal benefit in this case.

The patient recovered uneventfully, with the return of bowel function by postoperative day 3 and progressive dietary advancement. He was discharged on postoperative day 5 to a rehabilitation facility. Although he required additional rehabilitation for several weeks to regain full mobility, he eventually returned to independent living. At three-month and six-month follow-ups, the patient remained asymptomatic, with no clinical or radiological evidence of disease recurrence. His iron deficiency anemia had resolved, and laboratory markers, including CEA, remained within normal limits. He is scheduled for routine surveillance imaging and colonoscopic surveillance per standard guidelines.

## Discussion

Colorectal MC is an uncommon yet distinctive subtype of colorectal cancer, often exhibiting MMR deficiency and abundant lymphocytic infiltration [1,4]. Despite its high-grade appearance, MC may paradoxically carry a more favorable prognosis compared to poorly differentiated adenocarcinoma, possibly due to robust immune activation triggered by high MSI [2,4]. One-year and two-year survival rates of 93% and 74%, respectively, have been reported in MC [1,2]. However, MC poses significant diagnostic challenges when biopsies are limited and fail to capture the loss of characteristic MMR proteins (i.e., MLH1 and PMS2) and marked lymphocytic infiltration [3,4]. These hallmark findings highlight the importance of immunohistochemical profiling in distinguishing MC from poorly differentiated adenocarcinoma [1,5,6].

The management of MC remains controversial, especially regarding adjuvant therapy. While some studies suggest improved survival in MMR-deficient, MSI-high colorectal cancers, others caution that more prospective research is needed to confirm these observations [1,5,7]. In our case, the decision to omit chemotherapy was informed by the tumor’s MMR-deficient profile (loss of MLH1 and PMS2), stage IIA classification (T4N0M0), and the absence of additional high-risk features beyond lymphovascular invasion [7,8,9]. According to current guidelines, fluoropyrimidine-based adjuvant chemotherapy provides limited benefit in stage II MSI-high tumors unless significant adverse risk factors (e.g., perineural invasion or close margins) are present [10,11]. Our case aligns with these recommendations, though ongoing surveillance remains important given the rarity and variable behavior of MC.

The role of robotic surgery in the management of MC is currently not well established, and most evidence regarding enhanced precision, improved visualization, and shorter recovery times comes from broader studies in colorectal surgery rather than MC specifically [12]. In this case, robotic-assisted right hemicolectomy was selected based on patient- and tumor-related factors. The patient’s advanced age, preference for a minimally invasive approach, and the large tumor size influenced the decision to use a robotic platform rather than an open or standard laparoscopic technique. While data directly comparing robotic and laparoscopic outcomes for MC are scarce, certain benefits-such as superior instrument dexterity and stable three-dimensional visualization can be advantageous for complex resections or bulky tumors. Notably, robotic surgery did not appear to alter the patient’s oncologic outcome; instead, it may have contributed to a smoother recovery in an elderly patient who prioritized minimal invasiveness and a potentially faster postoperative course.

Close monitoring is crucial in MC due to the limited data on its natural history and the potential for recurrence. In this patient, periodic imaging and colonoscopic evaluations will be used to detect early recurrence, following the intervals recommended for similarly staged colorectal cancers. At six months postoperatively, the patient remains asymptomatic and free of disease. Further prospective studies or multicenter registries focused on MC are needed to clarify its long-term prognosis and to refine guidelines for both surgical approaches and adjuvant therapy in this rare but clinically significant tumor.

## Conclusions

This case highlights the diagnostic and management complexities of colorectal medullary carcinoma, highlighting the utility of comprehensive immunohistochemical analysis to differentiate MC from poorly differentiated adenocarcinoma. Although robotic-assisted right hemicolectomy may not confer a proven survival benefit over standard laparoscopic approaches, it can be a feasible and safe option for select patients - particularly the elderly - when surgical expertise is available. The omission of adjuvant therapy aligns with the tumor’s MMR deficiency and the current understanding that stage IIA MC may not benefit significantly from conventional chemotherapy. Finally, thorough follow-up is imperative in such rare malignancies to monitor for disease recurrence and to gather further data on optimal management strategies.

## References

[1] Al-Ishaq F, Al-Dhaheri M, Toffaha A, Awad S, Rizvi S, AbuNada M, Kurer M (2023). Colonic medullary carcinoma: an exceedingly rare type of colorectal malignancy: a case report and review of the literature. J Med Case Rep.

[2] Plummer PD, Yglesias B, Giuseppucci P (2023). Stage II medullary carcinoma of the colon: a surgery case report. Cureus.

[3] Fatima Z, Sharma P, Youssef B, Krishnan K (2021). Medullary carcinoma of the colon: a histopathologic challenge. Cureus.

[4] Winn B, Tavares R, Fanion J, Noble L, Gao J, Sabo E, Resnick MB (2009). Differentiating the undifferentiated: immunohistochemical profile of medullary carcinoma of the colon with an emphasis on intestinal differentiation. Hum Pathol.

[5] Thirunavukarasu P, Sathaiah M, Singla S (2010). Medullary carcinoma of the large intestine: a population based analysis. Int J Oncol.

[6] Schrag D, Rifas-Shiman S, Saltz L, Bach PB, Begg CB (2002). Adjuvant chemotherapy use for Medicare beneficiaries with stage II colon cancer. J Clin Oncol.

[7] Lanza G, Gafà R, Matteuzzi M, Santini A (1999). Medullary-type poorly differentiated adenocarcinoma of the large bowel: a distinct clinicopathologic entity characterized by microsatellite instability and improved survival. J Clin Oncol.

[8] Boland GM, Chang GJ, Haynes AB (2013). Association between adherence to National Comprehensive Cancer Network treatment guidelines and improved survival in patients with colon cancer. Cancer.

[9] Benson AB, Venook AP, Al-Hawary MM (2018). NCCN Guidelines insights: colon cancer, version 2.2018. J Natl Compr Canc Netw.

[10] Moertel CG, Fleming TR, Macdonald JS (1995). Intergroup study of fluorouracil plus levamisole as adjuvant therapy for stage II/Dukes' B2 colon cancer. J Clin Oncol.

[11] André T, Boni C, Mounedji-Boudiaf L (2004). Oxaliplatin, fluorouracil, and leucovorin as adjuvant treatment for colon cancer. N Engl J Med.

[12] Ravendran K, Abiola E, Balagumar K (2023). A review of robotic surgery in colorectal surgery. Cureus.

